# Suicidal and Dangerous Impulses in Insanity

**Published:** 1896-05-09

**Authors:** F. St. John Bullen

**Affiliations:** Late Assistant Medical Officer and Pathologist, West Riding Asylum, Wakefield


### Medical Progress and Hospital Clinics.

[*The Editor will be glad to receive offers of co-operation and contributions from members of the profession. All letters should be addressed to* The Editor, at the Office, 428, Strand, London, W.C.]

### SUICIDAL AND DANGEROUS IMPULSES IN INSANITY.

By F. St. John Bullen, M.R.C.S., Late Assistant Medical Officer and Pathologist, West Riding Asylum, Wakefield.

Although it is difficult to predict with certainty the liability to dangerous impulses in very many cases of insanity, it is allowable to indicate those in which they are especially to be looked for, and the relative strength of the tendency. For the sake of brevity, both suicidal and homicidal impulses will be considered together.

Self-destructive tendencies are by no means confined to melancholic states, nor aggression to maniacal. The proportion is roughly expressed, as regards the former, by the following statistics. Taking all forms of insanity, suicidal tendency is found in 25·8 males and 32 per cent. females, and of those suicidally inclined 59·6 per cent. were melancholic, 20 per cent. maniacal, 16 per cent. demented. As regards melancholia, from 60 to 70 per cent. are more or less suicidal, and about 30 per cent. actively so.

With the exception of simple depression, it is prudent to regard all cases of melancholia as potentially suicidal. There are some forms of insanity in which this tendency, or that of homicide, is greatly emphasized. To these allusion will now be made.

Attacks of insanity occurring at the epochs of life (puberty and adolescence, climacteric, senile), and at certain periods, viz., pregnancy, parturition, puerperal, lactational, the insanities of alcoholism, chronic brain atrophy, persistent brain instability, and epilepsy, need especial mention. The dangers of acute mania and melancholia, being obvious, may pass without mention. The pubescent and adolescent epoch in connection with mental disorder will be considered first. The normal psychology of this period of life explains the impulsive and hysterical tendencies usually shown, together with the markedly strong heredity present (quoted as 40 to 45 per cent. as compared with 29·5 for all forms of insanity). A large proportion of the cases show congenital moral defect; the tendency to the appearance of ancestral vices and persistence of developmental errors at this epoch are also notable features. In males the mental disorder is very often modified in type and aggravated in its explosive character by vicious habits. In the female this explosiveness is less frequent; the reproductive instincts are less awakened; moreover, the frequent presence of amenorrhœa and stuporose states have influence. On the whole, the type of mental disorder at adolescence is maniacal, in harmony with the egoism and motor developments proceeding.

Consequently is found maniacal excitement in three-quarters of the cases and aggressive conduct in about half, whilst suicidal tendencies are only present in one-fifth of the males, and probably in females a lesser proportion. But these suicidal tendencies are seldom persistent or acute; they partake of the general hysteric, quasi-dramatic character of the epoch. Briefly, then, the type is impulsive aggressive conduct, peculiarly accentuated and of a treacherous nature when vicious habits complicate the attack.

Whilst at the period of adolescence, most important additions are being made to the "ego," at the climacteric a large segment of organic consciousness, with its implicated group of higher evolved feelings, is withdrawn. The removal of so important a mass of sensations cannot fail to cause starvation of large areas of nerve-tissue, a damaged integrity of self-feeling, serious disturbance of mental equilibrium, with the result of impulsive tendencies from unopposed or uncontrolled ideas. Partial reductions in consciousness always tend to fulminant states, hence we look for explosive conduct, directed against self or others, in connection with the climacteric. Withal, there is a large element of indecision, corresponding to redistributions of nerve energy, which is unfavourable to persistent attempt at suicide or homicide.

It should be remembered that alcoholic habits possess a weighty influence in this connection; most dangerous impulse is to be feared therefrom of suicidal nature. In climacteric insanity depression upon the whole prevails, but notwithstanding this and the terrifying delusional concepts and religious despondency so often present, the number of actively suicidal patients is low. The percentage of suicidally-inclined is quoted as from 44 to 50 per cent.; but, as Clouston expresses it, though "a high proportion there is but a low intensity of suicidal impulse." However, in the worst cases suicidal attempts may be desperate.

A far less active disturbance is to be expected in man, from the more gradual decadence of reproductive activity, and the entrenchment of this period on the senile epoch.

In this last, acute maniacal and depressed states only exist in small proportion, but varied emotional storms accompany the progress of dementia. The tendency to impulsive acts, especially suicidal, is most to be dreaded when the senile reductions are associated with cardio-arterial degeneration and alcoholic habits. The ever-diminishing object-consciousness, the failure of contrasting ideas, together with the sudden emotional perturbations of pathological senescence, render the impulses born of painful organic consciousness peculiarly explosive and uncontrollable.

Insanity at certain periods now claims notice. Little need be said concerning that in connection with pregnancy; it is seldom met with as a pronounced psychosis. The few acute cases are characterised by intense agitation and strongly-marked suicidal proclivity; aggressiveness is also a feature.

Following on the nervous instability of the Gestation period, tension is at its height during parturition, when transient maniacal outburst may endanger the life of the offspring. Subsequently exhaustion, perhaps toxæmia, &c., together with the hereditary predisposition found in nearly half the cases of puerperal insanity, prepares us for the explosive form of this psychosis. This appears for the most part as acute mania of a very intense kind, characterised by extreme impulsiveness, suspicion and dread, and delusions and hallucinations of painful kind. Suicidal impulse is quoted as present in from 25 to 40 per cent. of cases, both in maniacal and melancholic states, whilst violent tendencies have been observed in nearly half the cases; particularly is the life of the child endangered.

Many cases which escape mental derangement at the puerperal period fall victims to the additional strain of lactation. The insanity of these two periods overlaps in character as in time of onset. The mental reduction in lactational insanity is less sudden and deep; and though acute maniacal agitation is a feature, furiously impulsive acts are not to be dreaded to the same extent as in puerperal insanity. Suicidal propensities are quoted as present in 32 to 40 per cent.; aggressive conduct in 59 per cent. Delusions and hallucinations, mostly of a painful type, are present in even a larger proportion than in the puerperal state. The former appear in nearly three-quarters of the cases. We pass on from dealing with these more or less explosive psychoses to a consideration of the very type of convulsive neuroses, viz., alcoholic insanity.

Bevan Lewis says: "There is in alcoholism an increase in the specific resistance of the afferent and efferent nerve fibres"; peripheral sensations are delayed and distorted, hence the lessened vividness of object-consciousness. Increased tension of muscular thought, a result of impeded efferent impulses, involves a rise of subject—consciousness and painful emotions with ideas of hostile environment prevail. With increasing dissolution of the "ego," volition becomes ever more paralysed and the motor resistance apparently augmented, producing still more gloomy and distrustful states of mind. Hallucinations, nearly always of harassing nature, determine impulsive acts out of these dispositions. Even the maniacal forms, which constitute more than half the cases, are not free from these suspicious moods. From the tendency of the unstable alcoholic's brain to discharge itself convulsively, and from the nature of the hallucinations, delusions, and disposition, most impulsive conduct, aggressive and suicidal, is engendered; thus violent proclivities are found in 68 per cent., self-destructive in 50 per cent. These inclinations only disappear when dementia is far advanced. There is one form of brain disease in which the acme of these impulsive propensities is reached, viz., chronic alcoholic cerebral atrophy. This, clinically, is chronic melancholia, with intellectual torpor and impulsive tendencies, associated with cardio-arterial degeneration, often renal, progressive motor enfeeblement, and paralysis; 66·6 of these cases are determinedly suicidal, and 83 per cent. dangerous. In pure hypochondriacal melancholia, suicidal attempts are rare; cases of sexual hypochondriasis in the male are said to be exceptions to this, and also that of chronic alcoholism. In melancholic stupor aggressive or suicidal impulse may be shown; such paroxysms may occur during the stupor, or more especially when this begins to pass off.

Another very impulsive form of mental disorder is Recurrent insanity (persistent mental instability). In this a strong hereditary influence is found; its subjects are throughout life peculiarly unstable, frequently of defective intellect and morale; in them criminal proclivities, alcoholic habits, and sexual perversions largely exist. The type of the attacks is subject to the usual modifying influences of causation, epoch, and period of life. Over half of these cases are highly dangerous to others, and about a third are suicidal. These cases of defective inhibition, when control is yet further removed by alcohol, are subject to an epileptic-like furore.

Space forbids more than brief mention of epileptic insanity. Two marked features of the disorder are the predominant self-consciousness and explosive tendencies. Hence arises the narrow egoism, fostering suspicion and hostile delusions, cherishing imaginary offences and schemes of revenge. From the sudden and intense mental reductions are born the impulsiveness of conduct which renders these patients the most dangerous of the insane community. Suicidal or homicidal outbursts may be met with in the intervals between fits, prompted by anger or delusion; during the furore subsequent to convulsions, either as a blind impulse, or from delusions existing at the time of the onset of the fit; lastly, during the period of epileptic automatism.

The impulses of the insane are largely determined by hallucinations and delusions. An opinion as to the influence they will possess in precipitating action can only be formed in the individual case. Their nature, persistence, effect on the patient's emotional condition, his strength of inhibitory power, and other numerous considerations will have to be entertained. The limits of this article have compelled scant allusion to, or omission of, many features of interest in connection with this subject; all that has been attempted is to place a few suggestive facts before those who are not especially conversant with insanity.

The statistics have been mainly gathered from the works of Bevan Lewis and Clouston.

